# Between welcome culture and border fence: A dataset on the European refugee crisis in German newspaper reports

**DOI:** 10.1007/s10579-023-09641-8

**Published:** 2023-02-13

**Authors:** Nico Blokker, André Blessing, Erenay Dayanik, Jonas Kuhn, Sebastian Padó, Gabriella Lapesa

**Affiliations:** 1 Research Center on Inequality and Social Policy, University of Bremen, Bremen, Germany; 2 Institute for Natural Language Processing, University of Stuttgart, Stuttgart, Germany

**Keywords:** Discourse Network Analysis, Policy debates, Annotation, Immigration

## Abstract

Newspaper reports provide a rich source of information on the unfolding of public debates, which can serve as basis for inquiry in political science. Such debates are often triggered by critical events, which attract public attention and incite the reactions of political actors: crisis sparks the debate. However, due to the challenges of reliable annotation and modeling, few large-scale datasets with high-quality annotation are available. This paper introduces *DebateNet2.0*, which traces the political discourse on the 2015 European refugee crisis in the German quality newspaper *taz*. The core units of our annotation are political claims (requests for specific actions to be taken) and the actors who advance them (politicians, parties, etc.). Our contribution is twofold. First, we document and release *DebateNet2.0* along with its companion R package, mardyR. Second, we outline and apply a Discourse Network Analysis (DNA) to *DebateNet2.0*, comparing two crucial moments of the policy debate on the “refugee crisis”: the migration flux through the Mediterranean in April/May and the one along the Balkan route in September/October. We guide the reader through the methods involved in constructing a discourse network from a newspaper, demonstrating that there is not one single discourse network for the German migration debate, but multiple ones, depending on the research question through the associated choices regarding political actors, policy fields and time spans.

## Introduction

In recent years, the topic of immigration has increasingly moved into the public eye. This is a development that is not least driven by the so-called European “refugee crisis” in 2015, which affected citizens at multiple levels (practically, but also emotionally) and thus kept both law makers and the media in suspense. Politicians and public figures responded to the growing numbers of refugees with rapidly changing policy proposals, which were reported and discussed in the media. Newspapers are an extremely valuable source for the empirical investigation of the effects of such crises on policy debates, because of the fine-grained representation they provide, both at the level of content (extensive, even redundant reports of the positions of politicians and parties) and at the level of time (multiple articles per day).

Newspaper corpora are therefore one of the primary sources used by *text-as-data* approaches within political science to study the dynamics of policy debates in order to understand coalition building and decision making processes. Typical research questions are: What are the driving actors and prominent issues of the debate? Which constellations herald turning points of the discussion? Will new coalitions emerge or old ones prevail? As these examples illustrate, the focus of this approach is generally on political actors and their positions on the specific policy issues [referred to as *political claims* in the literature, Koopmans and Statham (1999)]. Unfortunately, the text-as-data approach suffers from the lack of available datasets which are both large-scale and provide high-quality annotation, qualities that are needed in order to make robust generalizations.

**Fig. 1:**
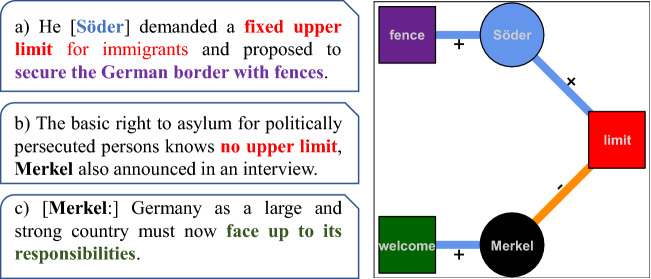
From text to networks: Textual spans containing political claims translated from articles in the German newspaper *taz* (left panel) mapped to the corresponding network representation (right panel). Circles indicate actors and squares claims. Edges express either support ($+$, blue) or opposition (−, orange). (Color figure online)

The work reported in this paper evolves around a dataset for this type of investigation, which we call *DebateNet2.0*,^1^ and which is released with this paper.^2^ It contains high quality annotation on the topic of immigration in Germany in 2015, based on a collection of articles from a quality newspaper: *die Tageszeitung (taz)*. The annotation targets claims (reported demands or propositions) made by political actors (politicians, parties, organizations, etc.) regarding specific actions to be taken within the heterogeneous policy field of immigration, covering aspects of, e.g., migration control, foreign policy, integration, solidarity, racism, and others. The leftmost panel of Fig. 1 illustrates an example of the annotation: the three textual spans contain claims attributed to two actors, Markus Söder and Angela Merkel. In the first span (a) Söder makes two claims: demanding to fix upper limits for immigrants (red highlight) and to build a border fence (purple highlight). In the second span (b), Merkel claims the opposite regarding the upper limit, and in (c) pushes for a so-called “refugees welcome” policy strategy (green).

Claims in *DebateNet* are labeled according to an annotation schema (codebook) defined by political science experts, containing approximately 100 categories and thus providing an abstract, but nevertheless extremely fine-grained, representation of the discourse. Such a fine-grained approach to annotation supports political scientists in gaining a deeper understanding of democratic decision making in the light of discourse dynamics.

The fine-grained annotation of actors and their claims described in the previous paragraph does not constitute an answer to the research questions about discourse dynamics we mentioned earlier, such as identifying actor coalitions or turning points in the discussion. What is needed is an abstract representation of the discourse and a methodology to mine the pattern of the discourse. We employ the Discourse Network Analysis (DNA) framework (Leifeld, 2016), a text-as-data methodology which has become increasingly popular in political science that brings together qualitative discourse analysis (QDA) and network analysis. DNA builds on the assumption that policy debates can be modeled as a bipartite network as the one depicted in the right panel of Fig. 1. The network contains two types of nodes: actors (round) and claim categories (square). The undirected edges between the claim categories and the actors encode the fact that the actor made a statement regarding the specific claim categories, along with the polarity of this statement. Although no edges are established between nodes of the same type, this representation permits the exploration of the status of specific nodes exactly by looking at its relations to the nodes of the other type. In our toy example, the upper limit claim is adequately captured as a (contested) one, as it allows us to contrast the two actors on the stage. One of the contributions of this paper, along with the release of *DebateNet2.0*, is a step-wise description of the workflow that turns textual data into discourse networks.

Along with *DebateNet2.0*, we also release its companion R package, mardyR^3^. The focus of this package is to visualize discourse networks derived from the annotations: it supports both the basic querying and subsetting of the annotation (e.g., all the claims made by Angela Merkel on a certain time span) as well as the visualization of the discourse networks, enabling the user to trace policy debates by linking actors to their claims in textual sources, with the ultimate goal of capturing a comprehensive picture of the discourse over time. We demonstrate the different insights that can be gained by parametrizing the network at different levels: focusing on actors vs. on specific claim categories vs. on actor/claims to carry out comparisons between time slices. In addition, both (queried) annotations and networks can be exported for downstream analysis (e.g., with corpus linguistics methods).

In the second part of the paper, we discuss the impact of our research for political science research. We do so first by means of a DNA case study, targeted at the characterization of the crisis/debate interplay. In particular, we focus on two stages of the refugee crisis: Spring 2015, when a large number of migrants tried to reach Europe via the Mediterranean, resulting in many lives being lost at sea; and fall 2015, when the migration flux followed the Balkan route, resulting in large number of refugees at the European borders and increasingly restrictionist migration policies. Beyond the results of our analysis, this case study also serves as an illustration of the analysis of *DebateNet2.0* with mardyR and as a guideline for the interpretation of the output of the queries of mardyR on *DebateNet2.0*. Next, to further characterize the usefulness of our resource for political science research, we discuss further research carried out on *DebateNet*.

*Contributions.* The contributions of this paper comprise multiple levels. At the level of resources, we document and release *DebateNet2.0* and mardyR. *DebateNet2.0* addresses the desiderata for a large-scale high-quality policy debate dataset, namely (a) an annotation schema developed by domain experts, capable of capturing the fine-grained nature of the policy making process; (b) the availability of annotation layers which allow for a meaningful parametrization of the queries (e.g., for political actors, mapping of different mentions to a canonical form, and, ideally, to a party: “Die Kanzlerin”, “Merkel” $\rightarrow$ Angela Merkel $\rightarrow$ CDU); (c) the availability of tools and theoretically motivated case studies which can serve as guidelines for the visualization and a meaningful interpretation of the results of the query.

At the experimental level, we discuss the results of our case study comparing the discourse network representations of two crucial stages of the refugee crisis in Germany.

At the level of methods, we provide a step-by-step illustration of the process of deriving discourse network representations from texts and discuss the conceptual and practical issues related to this process. Additionally, we provide the reader with guidelines for the use of our dataset and for the interpretation of the output of its analysis. We consider these guidelines to be an additional resource we contribute together with *DebateNet2.0* and mardyR.

We believe that our contributions are relevant both for researchers interested in the immigration topic—which is the substantive focus of our investigation—and to the methodically oriented general NLP and political science communities. From an NLP perspective, the construction of discourse networks from large amounts of raw text is very challenging and has thus the potential to trigger progress in many subtasks. At the same time, political debates are a relatively stable, well-behaved type of debate which can serve as a blueprint to structure the understanding of how similar debates unfold under less controlled circumstances, such as in social media.

From the perspective of political science, we take a further step in improving the understanding of highly dynamic debates on the heels of a (perceived) crisis. With the help of NLP, the dataset provided here builds the foundation to scale up the annotation of political text both with regard to text types (genres) and scope (topic and volume).

### Plan of the paper

The paper is structured as follows: Sect. 2 provides background for the Discourse Network Analysis framework, along with a summary of related work within the NLP literature. Section 3 describes *DebateNet2.0* in all details, from the selection of the source data, to the annotation, further processing, and quantitative analysis. Section 4 is dedicated to mardyR. In Sect. 5, we assess the impact of our resource on political science research by means of a case study, the comparison of the discourse networks from mid-April to May (Mediterranean route) and late September to mid-November (Balkan route), and with a summary of further studies conducted on the dataset. Section 6 concludes the paper by summarizing its findings and contributions. We outline further modeling work we have carried out on the dataset and identify perspectives for future work.

## Background: claims analysis, discourse networks, and NLP

Understanding the structure and evolution of political debates is essential for understanding democratic decision making, and is therefore of central interest to political science (de Wilde, 2011; Zürn, 2014; Haunss & Hofmann, 2015).

Democratic decision making can broadly follow two logics, one of which is a *technocratic* mode, where decisions are taken by administrative staff and field-specific experts. We focus on the second type of decision making, the *politicized* mode, which proceeds through programmatic statements (Schmidt & Radaelli, 2004) and political debates. While there is no general theory about mechanisms driving political discourse, there seems to be general agreement that the formation and evolution of discourse coalitions is a core mechanism (Hajer, 1993; Sabatier & Weible, 2007) and that change in these coalitions is influenced by external events and by the discourse itself (Leifeld, 2016).

One promising way to gain insight into such discourse dynamics in an empirically robust fashion, based on widely available newspaper corpora, combines political claims analysis (Koopmans & Statham, 1999) with *discourse network analysis* (Leifeld & Haunss, 2012). The unit of analysis is the *claim*, that is, a demand, proposal, or criticism that is *supported* or *opposed* by an *actor* (a person or a group of persons) and can be *categorized* with regard to its contribution to the debate at hand. Crucially, not all statements concerning the topic are to be considered claims, but only those which propose or oppose a specific action (e.g., giving empty flat to refugees). Claims and the actors who put them forward are represented together in a bipartite *affiliation network*, as shown above in Fig. 1. Discourse coalitions can then be obtained by *projecting* the affiliation network on the actor side (i.e., constructing a network with only actor nodes, which are linked if they take similar stances towards the claims, cf. Leifeld (2016) and Sect. 3.4). Conversely, a projection on the concept side yields the argumentative clusters present in the debate.

Clearly, manual annotation of claims and claim-actor relations is a resource intensive process. It it therefore natural to ask if natural language processing (NLP) can help: What are the potentials, limitations, and the practical issues of applying NLP to the automatic construction of discourse networks?

At a general level, the NLP take on debate modeling can build on the insights from argumentation mining and subjectivity analysis (Peldszus & Stede, 2013; Ceron et al., 2014; Swanson et al., 2015; Stab & Gurevych, 2017; Vilares & He, 2017). An ideal NLP tool would automatically identify the actors and their contributions to the debate, and analyze such contributions at a structural level (identifying argumentative structure in their statements), at a semantic level (classifying statements into relevant categories), and at a pragmatic level (detecting the polarity of the statements).

Based on our experience (Padó et al., 2019), however, we argue that this task cannot be automated completely at the present time, at least not if the goal is to maintain the levels of (fine) granularity and quality which are necessary to answer the substantive research questions posed by political science. What proved successful for us is instead the integration of manual annotation and NLP methods (Blessing et al., 2019) into a semi-automatic procedure that speeds up the manual work by providing intelligent proposals, efficient annotation interfaces, and adding automatic predictions as pre-annotation that are clearly labeled as such. We find that this approach scales up with manageable loss in granularity and quality and can serve as an example of successful *mixed methods* that form the interface between big data on one side and the humanities and social sciences on the other (Kuhn, 2019).

## DebateNet2.0

We now describe the steps that we took to transform a set of newspaper articles into a discourse network representation for the corresponding debate. Our workflow proceeds in the following steps:

1. Selection of the textual sources, at the level of corpus (Sect. 3.1) and of the articles to be annotated (Sect. 3.2);
2. Annotation (Sect. 3.3), at multiple layers: claim identification and classification, actor identification, claim attribution (establishment of an edge between an actor and a claim), assignment of polarity (actor is pro vs. con the claim), assignment of a date to the claim/actor pair (which may be different from the date of the article);
3. Extraction and querying of discourse networks from the annotation (Sect. 3.4).^4^

### Source corpus

The source corpus consists of newspaper articles from the German quality newspaper *die Tageszeitung (taz)* spanning the entire year of 2015. We choose newspaper articles for three main reasons.

First, newspaper articles constitute an elite discourse that affects democratic decision making (Schneider et al., 2007; Page, 1996): they aggregate claims of multiple actors while simultaneously filtering content less relevant to the discussion at hand. We are aware that this gatekeeper function (White, 1950) of the media is a double-edged sword, as the selection and the framing of the reported news can vary significantly in relation to the editorial line of the newspaper (Hagen, 1993), and is also manipulated at higher levels (*agenda-setting*). We chose *taz*, which, while left-leaning, is nevertheless considered a quality newspaper, meaning that it is reasonable to assume that claims form central actors from the entire political spectrum are reported.^5^

Second, newspapers offer a broader overview of the discourse than most other political texts, such as party manifestos or parliamentary speeches. Hence they combine partisan, institutional and civic representatives on the level of individual actors and organizations.

Third, this wide range of political entities enables and requires a fine-grained analysis of the issue at hand, that translates well into the use of methods such as Discourse Network Analysis (Leifeld, 2016). Such breadth could be targeted as well by gathering social media activity of members of parliament: the focus on policy-making would be, however, definitely less sharp and retrieving the claims would be more challenging (as the journalists both filter and highlight the relevant information).

### Article selection

**Fig. 2:**
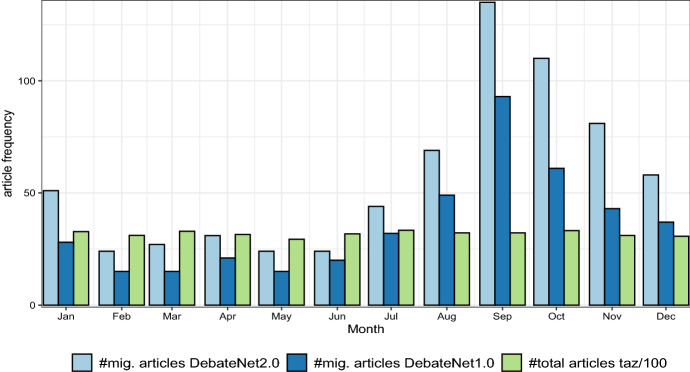
Number of articles on migration and total articles per month. (Color figure online)

The entire corpus contains around 38,000 articles from which roughly 700 contained claims on migration politics in Germany and were therefore selected for manual annotation. More specifically, we defined a set of seed keywords connected to the topic (asylum, refugee, migrant/migration, etc.) and German politics. Subsequently, we trained a binary classifier (Joulin et al., 2016), optimized on recall, on the articles found in this fashion to identify articles not covered by the keyword-based approach. Additionally, annotators then flagged false positive articles for being off topic. An additional criterion was applied to the subsequently presented dataset: Articles were only included when they contained at least one political claim. Figure 2 illustrates two developments. First, while the total number of articles (shown in green) published remains relatively stable over the months (averaging around 3200 articles, sd = 119), the number of articles covering migration-related topics (shown in blue) fluctuates considerably (mean = 37, sd = 57). Especially between August and December, there is a substantial increase in migration-relevant articles, peaking in September. This indicates that the newspaper placed more relative attention in this time-frame on the issue of migration at the expense of other topics. Secondly, the difference in numbers between *DebateNet1.0* and *DebateNet2.0* (light and dark blue, respectively) appears relatively uniformly distributed across months. Therefore, the previous release can be seen as a random sample of the complete dataset, from which similar results might carry over. Note however that this does not imply that the content of *DebateNet1.0* is also a representative sample of *DebateNet2.0* (cf. Sect. 5.1).^6^

### Annotation

As mentioned in the introduction, our units of observation are textual spans that realize the political claims and actors which are later to form the nodes of the bipartite discourse network, as well as the relations between claims and actors that will form the edges of the network. Our annotation therefore starts from raw text and proceeds in multiple steps:

*Step 1: Claim identification*. First, the annotators have to identify the textual spans containing claims. As shown in our examples in Figure 1, claim-bearing textual spans do not necessarily coincide with sentences: they can be a subpart of a sentence, or span beyond the sentence boundary. Discourse network-wise, this corresponds to the identification of the claim node (squares in Figure 1). The claim node is further annotated with the document ID.

*Step 2: Claim classification*. The textual spans identified above are assigned to one or more claim categories from the annotation scheme (called *codebook* in political science), which structures the annotation process. Categories and annotations are moving targets, they traverse a hermeneutic cycle during the annotation process: the aptitude of individual claim categories is constantly reviewed, new categories are adopted, outdated categories revised, overlapping categories merged. All this requires a close cooperation between experts and trained annotators in order to maximize inter-coder reliability.

**Table 1:** High-level categories across annotations

Major	Description	Frequency	Percentage
100	Controlling migration	992	22
200	Residency	630	14
300	Integration	386	9
400	Domestic security	154	3
500	Foreign Policy	711	16
600	Economy + Labor Market	153	3
700	Society	740	17
800	Procedures	651	15

Overall, 110 detailed sub-categories exist that are grouped into 8 high-level classes shown in Table 1. Besides fine-grained categories, the codebook also contains descriptions and defining examples providing guidance to our annotators (see Table 8 in “Appendix A” for an example; the codebook can be downloaded from http://hdl.handle.net/11022/1007-0000-0007-DB07-B). Claims are unevenly distributed across classes, reflecting the multiple foci of the discussion at hand. Thus, the claim nodes identified at the previous step are now characterized by two annotation layers, namely the fine-grained and the coarse-grained categories.

*Step 3: Date assignment*. The claim is assigned a date, which is by default the day preceding the publication of the article. It is the annotator’s task to reconstruct the claim date, based on textual information. The assigned date is a further layer in the annotation of the claim nodes.

*Step 4: Actor identification and mapping*. The annotators identify the strings corresponding to actor mentions (e.g., “Angela Merkel”, “Die Kanzlerin”, “Frau Merkel”). Note that a single claim can be attributed to more than one actor, and actors can be mentioned inside or outside the textual span. Discourse network-wise, this step corresponds to the identification of the actor node (circles in Fig. 1). The actor nodes are further annotated with: (a) named entity (PER vs. ORG), (b) party,^7^ for the politicians, (c) mapping of the actor mention to a canonical name which serves as a unique identifier of the actor in the dataset.

**Table 2:** Actor mapping

	Pattern	Example 1	Example 2
1	String	A. Merkel	Mehrere Ministerpräsidenten
2	Spelling	Angelika Merkal	Mehrere Ministerprasidenten
3	Parts	Merkel	Ministerpräsidenten
4	Synonym	Kanzlerin	Länder-Chefs
5	Canonical name	Angela Merkel	Mehrere Ministerpräsidenten
6	Surrogate	Spokesperson	Spokespersons
7	Pars pro toto	Bundesregierung	
8	Totum pro parte		Alle Bundesländer
9	Possible name	Bundesregierung	List of all names

Table 2 contains two examples highlighting difficulties that arise during the process of actor mapping. While Example 1 refers to the actor by name, Example 2 points to the institutional role/position of the actors instead. The top half of the table (rows 1–5) displays a set of operations which fall under the general label of *lexical mappings*. Broadly speaking, the goal of lexical mapping is to find the canonical name of the actor. This implies unifying different notations, rectifying spelling errors, completing names, and resolving synonyms. At the end of this process, “Angela Merkel” emerges as the canonical name in Example 1 and “multiple prime minister” in Example 2. While the former is a proper actor, the latter obviously still needs to be resolved to individual ministers. But what if we simply cannot know which prime ministers are being referred to here? In this case, we can either stick with the somewhat unsatisfying canonical name that we found or settle on a set of mapping rules. For instance, we could map the prime ministers as heads of their corresponding states to the group-actor “Bundesländer” representing the states in Germany.

The bottom half of Table 2 (rows 6–9) illustrates even more problematic cases, in which mapping requires assuming considerations that are even harder to formulate. This is the case of spokespersons or institutional actors that represent another actor. Does the spokesperson of the federal government also speak for Angela Merkel as the chancellor and leader of the government? If so, we blur the line between the lexical and the semantic level. If not, we might lose many claims that are concerned with the daily business of running a country. How to resolve this trade-off is a matter of the research question at hand. Consequently, *DebateNet2.0* contains only conservative lexical mapping rules, in line with its design to be a resource across disciplines. More specific mapping rules can be added for individual use cases.

*Steps 5 and 6: Claim attribution and polarity assignment*. The claims are now explicitly linked to the relevant actor. Discourse network-wise, this step corresponds to establishing an edge between the claim and actor nodes. The last step is to assign a polarity (support/opposition) to the attribution edge.

### From texts to discourse networks

On the basis of the annotation described in the previous section, we can already carry out a rich descriptive statistical analysis to gain qualitative as well as quantitative insights. However, to obtain a discourse network, two more steps steps are routinely carried out and described directly below. Depending on the research question, it may be advisable to further transform the data or impose temporal constraints (Leifeld, 2016). Some possible scenarios are covered in Sect. 5.1.^8^

**Fig. 3:**
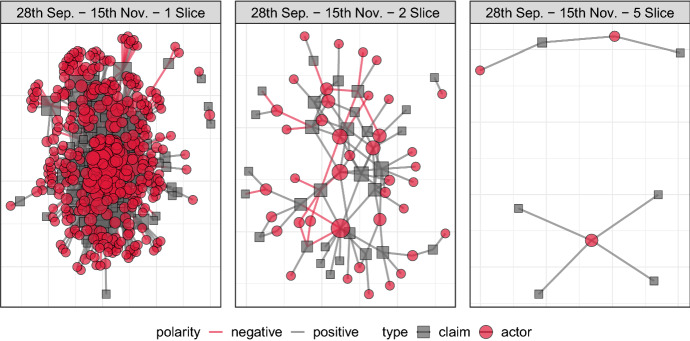
Defining the core network: 1-slice (left), 2-slice (middle), 5-slice (right)

*Step 7: Aggregation*. Aggregation concerns the unification of individual annotations. It is, again, parametrized by the requirement of the research question, and generally involves multiple steps. The typical first step is to aggregate across time-windows, and perform a first filtering on the actor/claim pairs which occur at least *m* times, a typical value of *m* being 2. This step builds on the assumption that, given the redundancy of the reports, hapaxes at an actor/claim pair can be considered spurious and can be trimmed away without losing relevant information. The resulting network is referred to as the *core* network (Scott, 2000, pp. 112–113) or, more technically, as a *m-slice* of the complete network (de Nooy et al., 2005, p 98). The intuition behind the core network is to extract only those nodes and edges that are central to the debate and filter out potential noise. For purely illustrative purposes, we display in Fig. 3 the 1-slice (raw), 2-slice and 5-slice network for the same time period (circles are actors, squares are claims). The 1-slice network is extremely dense, contains all of the many actors and claims that appear at least once, and is therefore often difficult to interpret. In contrast, in the 5-slice network very little of the structure of the debate is left (indeed, actor–claim pairs are rarely mentioned at least 5 times on different days within the specified time-window). In the following sections, we will adopt a 2-slice representation, which generally strikes an appropriate balance between relevant information and comprehensiveness.

After the hapax claim/nodes pairs are filtered away on a day-base, claims are typically aggregated further based on annotation properties, e.g., aggregation on a monthly basis, or on the basis of a claim category, or actor, or a combination of them.

**Fig. 4:**
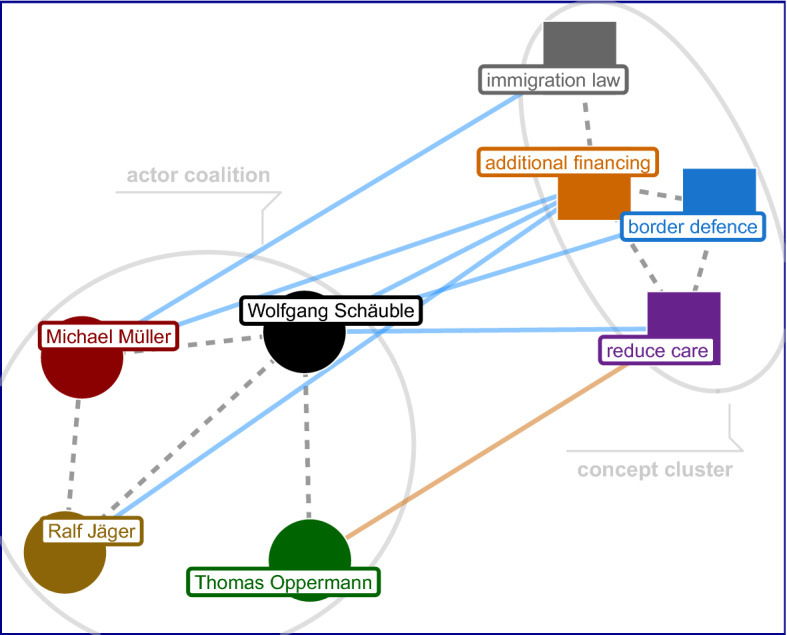
(Bipartite) Affiliation network with claims and actors as two types of vertices in the network (4 actors 4 claims). Edges are colored according to their weight. Dashed and gray lines represent the projected variants of the one-mode networks; adapted from (Janning et al., 2009, p 71)

*Step 8: Projection*. A transformation that is often useful after aggregation is projection, where the actor-claim bipartite network is transformed into an actor-only or claim-only network, with the connectivity of the resulting network mirroring similar contexts in the bipartite network (Leifeld, 2016). Thus, the result can be interpreted in terms of conceptual similarity among actors, or among claims, respectively. Figure 4 illustrates how the projection of the actor nodes on the claim nodes gives rise to so-called concept clusters, while the opposite operation identifies issue communities from which actor coalitions—structured according to their respective stances (support or opposition)—may start to emerge.

### Descriptive statistics of *DebateNet 2.0*

In this section we provide descriptive statistics for *DebateNet 2.0*, at the level of actors, claims, and textual spans. This section is thus not concerned with Discourse Network Analysis which will be taken up again in Sect. 5.1.

#### Annotation details

*DebateNet 2.0* is in reality the result of going several times through steps 1–8 as defined above, but could in principle be archived in one single pass. We recruited annotators with domain expertise from the student body of political and social science or adjacent programs. After the students carry out their annotation, the results were adjudicated in a subsequent step by two experts and are merged into a so-called gold standard. In an earlier study, we determined the inter-coder reliability among students for claim identification to be $\kappa =0.58$ (Padó et al., 2019). The additional step adjudication step should improve the reliability further. The resulting dataset contains 3442 text passages, corresponding to 4417 individual claims (text spans may be assigned to multiple claim categories and actors).

**Table 3:** Frequent actors in the migration debate 2015 (after actor mapping)

Actors	
Name	Freq
Angela Merkel (federal chancellor)	247
Thomas de Maizière (minister of the interior)	162
Bundesregierung (federal government)	152
CSU (party)	86
Horst Seehofer (CSU chairman)	79
SPD (party)	78
EU (European Union)	77
Sigmar Gabriel (minister of foreign affairs)	68
Grüne (party)	60
Jean-Claude Juncker (president of EU commission)	57

#### Actor level statistics

Table 3 displays the 10 most frequent actors of the entire year. Unsurprisingly, the most prominent actor of the migration crisis in Germany is chancellor Angela Merkel, followed by minister of the interior Thomas de Maizière, and, as institutional actor, the federal government (“Bundesregierung”). Other relevant actors include Merkel’s political antagonist in the migration debate and chairman of the Christian Democratic Union (CSU), Horst Seehofer, Minister of Foreign Affairs, Sigmar Gabriel, and the President of the European Commission, Jean-Claude Juncker.

#### Claim-level statistics

Recall that Table 1 describes the distribution of higher-level-categories across the dataset. The most frequent category is “controlling migration”, which contains demands and proposals concerned with regulating immigration (border controls, upper limit, asylum law, etc.). Related and also prominent is “foreign policy” (e.g. EU-wide quota, international solutions). Other categories are “society” that deals with humanitarian and cultural aspects (human rights, Christian values) and “residency”, mostly concerned with the accommodation of migrants. Least frequent are “domestic security” and “economy + labor market” which are further downstream of the acute (perceived) crisis situation. Although, it stands to reason that both gained momentum after 2015 and are more present during the 2016 discussion (not covered in our dataset). A special, less topical category is “procedures” that often appears in combination with other categories (additional funding, transparency, etc.).

Tables 4 and 5 provide a more detailed breakdown, displaying the most frequent positive and negative sub-categories of 2015, respectively. EU solution (501), a European-wide quota for refugees, is the most often used claim with a positive polarity. Followed by calls for more funding (805) and an upper limit (102). Frequent claim categories with negative polarity include calls to oppose xenophobia (703), right wing radicalism (709), and the current immigration policies (190). An EU solution is also high on the list, indicating that this is a polarizing and contested claim category.

**Table 4:** Frequent positive claims

Positive claims			
Code	Claim category	Frequency	Total (incl. neg. Claims)
501	EU solution (quotas for refugees)	237	310
805	Additional financing	147	155
102	Ceiling/upper limit	129	152
812	Fast/accelerated procedure	124	132
207	Deportations	112	140
504	Safe country of origin	112	153
105	Border controls	103	126
705	Refugees welcome	94	107
309	Care (medical, financial,...)	87	119
104	Isolation/immigration stop	86	128

**Table 5:** Frequent negative claims

Negative claims			
Code	Claim category	Frequency	Total (incl. pos. Claims)
703	Xenophobia	129	161
709	Right-wing radicalism	86	98
190	Current migration policy	73	95
501	EU solution (quotas for refugees)	73	310
202	Refugee accommodation	56	79
110	Asylum law	49	98
711	Islam	47	63
104	Isolation/immigration stop	42	128
504	Safe country of origin	41	153
401	Violence against migrants	36	44

**Fig. 5:**
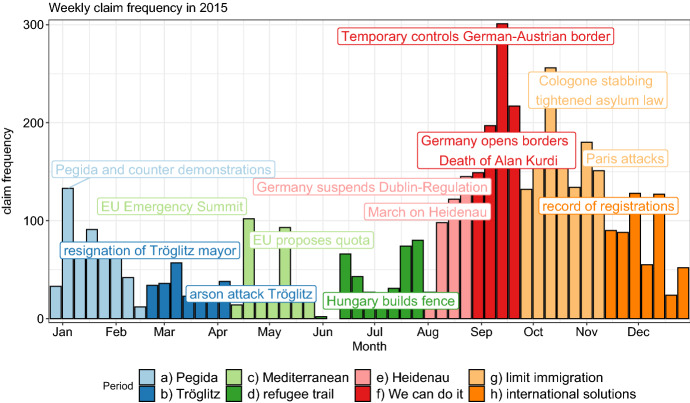
Timeline of the “refugee crisis” in Germany

#### Timeline

Figure 5 showcases the number of resulting claims over the course of 2015. The graph displays the weekly number of claims in 2015 roughly divided into time periods named after prominent events (modified after Haller, 2017,9).^10^ This is not meant to be a full-fledged analysis, but a rough classification that guides future examinations. The distribution of claims over weeks matches the distribution of articles shown in Fig. 2, with distinct peaks in September and October.

Each phase transition is a natural way to separate the debate into fragments that in turn can be analyzed in depth separately or in combination with other periods. For instance, with the numbers of refugees trying to escape the war in Syria via the Mediterranean the discussion about the necessity of sea rescues and efforts to fight smugglers flare up in April. A debate less critical by the time the “Balkan Route” gains prominence. Obviously, there is causal connection between relocating the points of entry into the European Union but the focus of the discussion has shifted to other policies to address the new developments (e.g. the Hungarian border fence “Grenzzaun”).

#### Text-level statistics: keyword analysis

An alternative way to explore and analyze the data provided in *DebateNet2.0* is using keywords. This approach builds on the assumption that changes in policies are accompanied by a different vocabulary to express them. In other words, are these changes reflected in a change of word distributions? To extract the keywords, we used the popular YAKE system (Campos et al., 2018) with standard settings. YAKE’s keyword extraction approach combines word frequency information with finer-grained features related to the position of a candidate keyword in the sentences in which it occurs, as well as with dispersion scores (at the sentence level and at the level of the different contexts of occurrence). For each annotated text passage, we extracted two keywords with YAKE. We then aggregate the keywords per month, ranking them according to frequency. Table 6 (p 17) displays the most frequent keywords for the month April (representing the *Mediterranean* period) and October (representing the *Balkan route* period). As expected, the word “Seenotrettung” (emergency sea rescue) and “Schlepper” (smugglers) are most frequent in April along with “Bundesregierung” (federal government). The latter also occurs frequently in October, together with all governmental parties (CDU/CSU and SPD) and high ranking government officials. As for claims, keywords indicating restrictionst policies rank highly in October, such as “Grenze/Grenzen” (border), “Transitzonen” (transit zones), and “Abschottung” (isolation).

**Table 6:** Most frequent keywords in April and October

April		October	
Keyword	Freq	Keyword	Freq
Bundesregierung (fed. government)	8	Spd (SPD)	20
Seenotrettung (sea rescue)	6	Flüchtlinge (refugees)	19
Cdu (CDU)	5	Cdu (CDU)	18
Maizière (Maizière)	5	Merkel (Merkel)	17
Europa (Europe)	4	Deutschland (Germany)	13
Schlepper (smugglers)	4	CSU (CSU)	12
Spd (SPD)	4	Bundesregierung (fed. government)	11
Asylbewerbern (asylum seekers)	3	Regierung (government)	10
Aktivisten (activists)	2	Flüchtlingen (refugees)	9
Asyl (asylum)	2	Seehofer (Seehofer)	8
Behandlung (treatment)	2	Transitzonen (transit zones)	8
Bitte (request)	2	Grenze (Border)	7
Bleiberecht (Right of residence)	2	Oktober (October)	7
Bundesamt (federal office)	2	Abschottung (isolation)	6
Bundesländern (states)	2	Grenzen (borders)	6
Bündnis (alliance)	2	Kanzlerin (chancellor)	6
Fähren (ferries)	2	Land (country)	6
Flüchtlinge (refugees)	2	Maizière (Maizière)	6
Rettungsmission (rescue mission)	2	Türkei (Turkey)	6

Note that we hand-picked the keywords in case there was a tie in frequencies. For October we present all keywords with a frequency above 5 while in April this threshold is 2

These findings demonstrate that the keyword lists can provide a general idea about the topics of discussion and the most central actors involved. Yet, it is not possible to make inferences regarding the relation between different actors and claims on this basis. This motivates our use, in the next sections, of discourse networks. We expect that they not only mirror these developments, but also shed some light onto relational aspects and the decision making along the process with it.

**Fig. 6:**
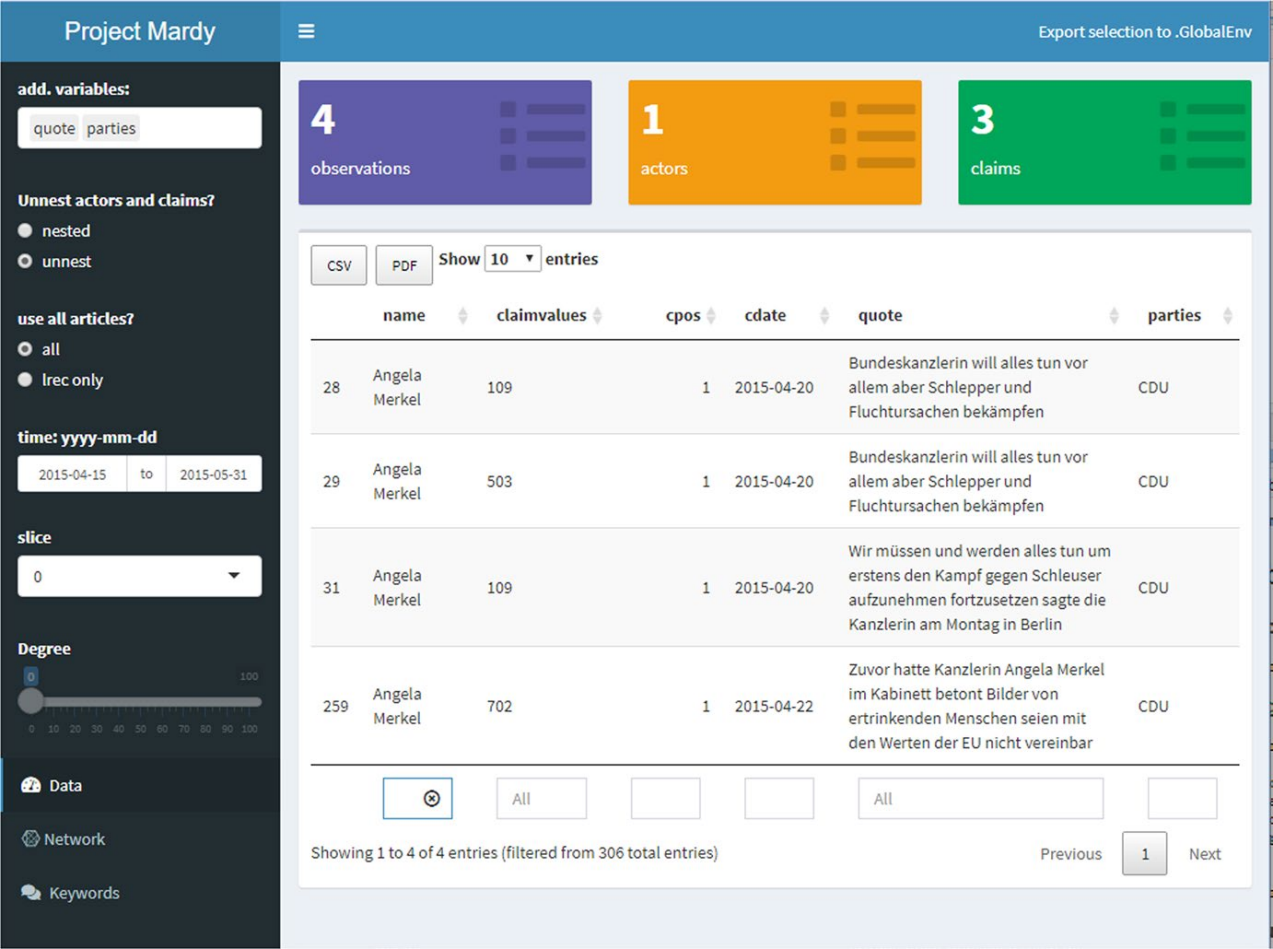
mardyR—dashboard

## Visualization with **mardyR**

In order to quickly inspect the DebateNet dataset and replicate the network construction steps described in Sect. 3 with different parametrizations, as well as to obtain a visualization of discourse networks (Sect. 5), we developed a companion software package in R, mardyR. The package offers an interactive, browser-based web application in the form of a dashboard built around the shiny^11^ framework (Chang et al., 2020; Chang & Borges Ribeiro, 2018).^12^

### Actor-level aggregation

**Fig. 7:**
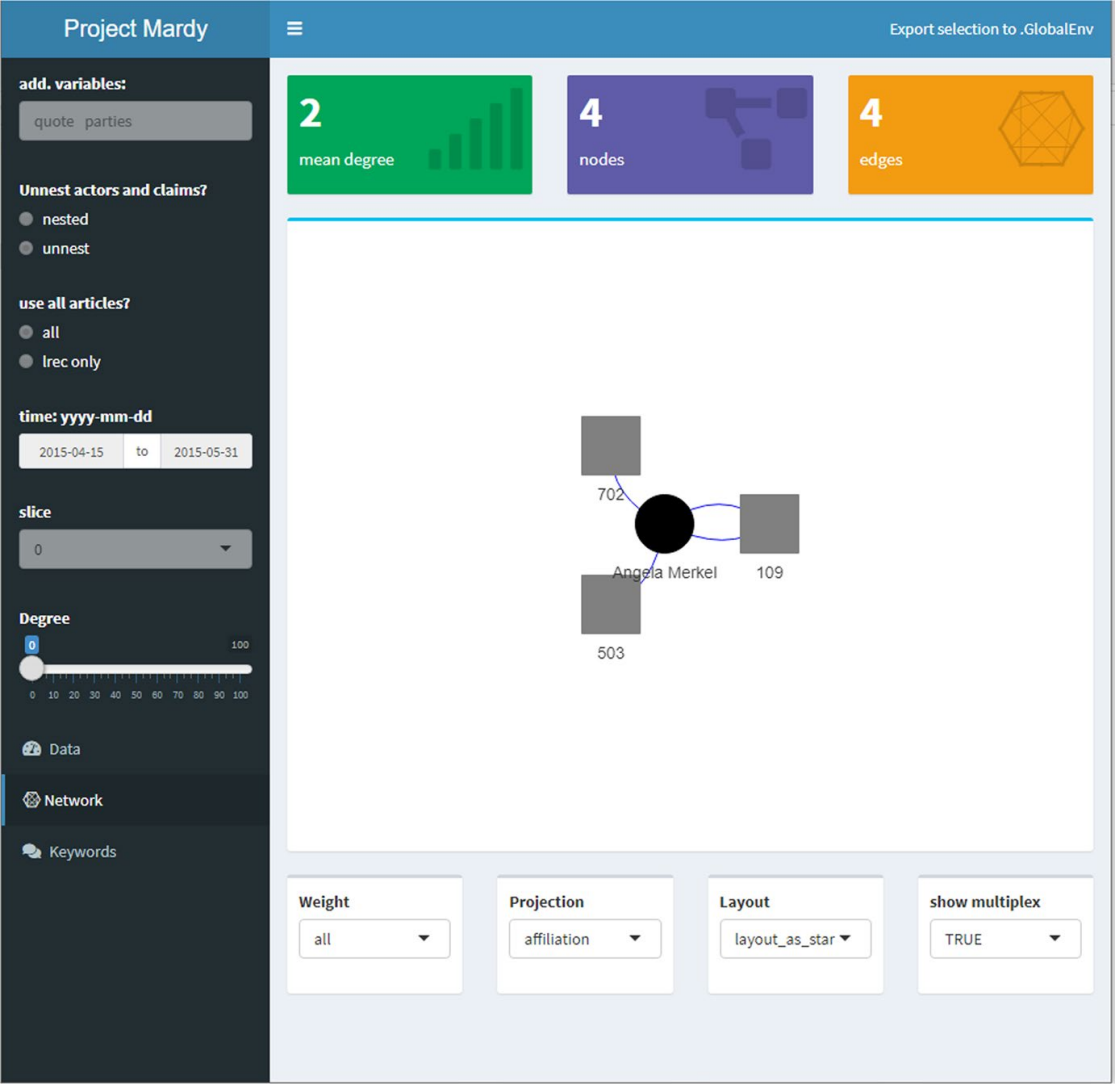
Spring: Ego-network Merkel

For demonstration purposes, we use this application to trace the involvement of chancellor Angela Merkel over the course of the debate. We selected the time-windows from Fig. 5, corresponding to “Mediterranean” and “Limit Immigration”). Figure 6 displays all claims by Merkel as reported in the *taz* from 15th April to 31st May. The entries of the dataset are presented in the right panel of Fig. 6 while the left panel contains configuration options, e.g., select variables, apply time-frame, choose which release of the dataset to use. In this period, she stresses the importance of humanitarian rights (702) and proposes to fight smugglers (twice, 109) as well to combat causes of flight (503). The corresponding network is displayed in Fig. 7 (p 18), with Merkel as round node surrounded by and linked to three square nodes representing claim categories. Overall, the resulting network consists of four nodes and four edges with a fairly low average degree centrality of 2 (measured as the average number of edges connected to a node, Wasserman & Faust, 1994, p 100).

**Fig. 8:**
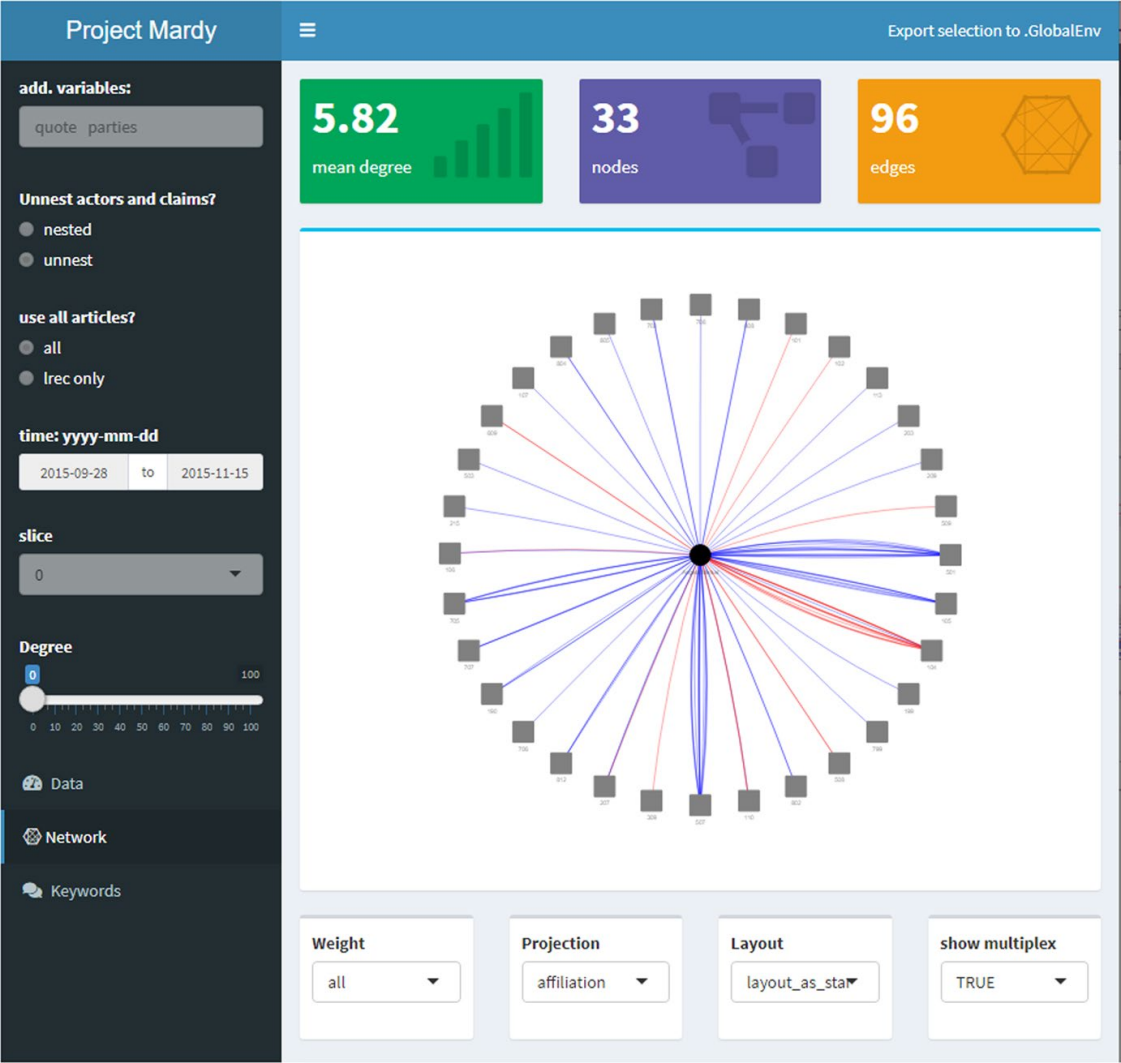
Fall: Ego-network Merkel

In contrast, Fig. 8 (p 19) makes it apparent how much more involved she became during the later period of the year from 28th September to 15th November. Here, 96 claims made by Merkel are reported in the newspaper, which translate into 32 distinct claim categories she either supports (blue edges) or opposes (red edges).

### Claim-level aggregation

**Fig. 9:**
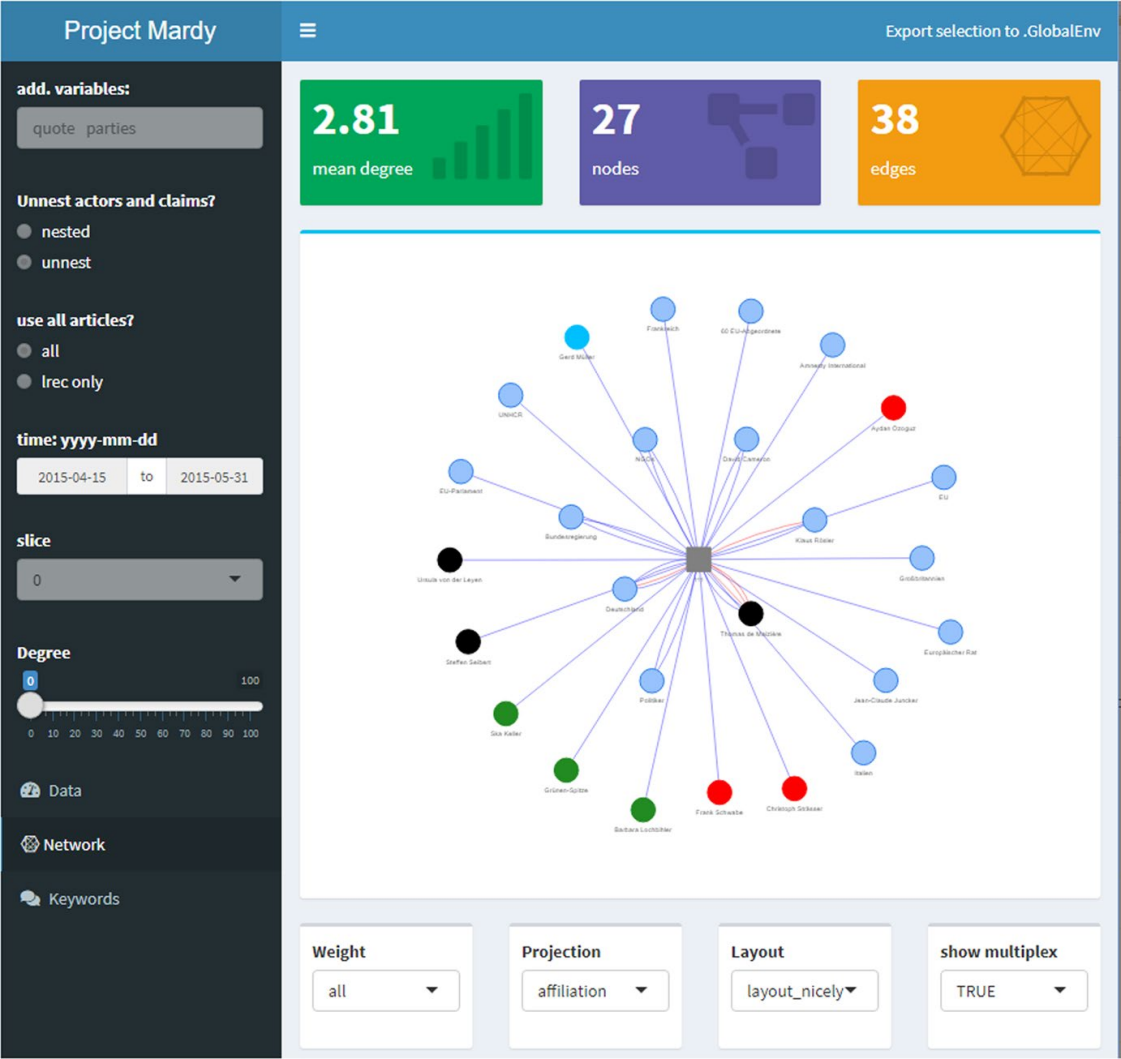
Spring: Ego network sea rescue

Similarly, one can trace the usage of different claim categories over time or cross-sectionally. In Fig. 9 (p 19) all instances of the claim “sea rescue” (111) from the first time period are displayed and linked to the corresponding actors (colors indicate party affiliation). The overwhelming majority supports the claim of sea rescue missions. Therefore, from a purely structural standpoint, it is even more surprising that this claim does not appear in the second time period at all. Instead of 111 other claims have emerged and gained popularity.

Overall, this goes to demonstrate the dyadic relation between actors and claims and vice versa. However, the real strength of social networks comes to light when the whole network with all its interrelations is considered simultaneously. Arguably, this is what captures the essence of the debate.^13^

## Impact

In this section, we demonstrate the potential impact of *DebateNet2.0* (and comparable resources) on political science research, in two steps.

First, in Sect. 5.1, we talk the reader through a concrete case-study: we compare two distinct time periods of 2015, spring and fall, corresponding to two phases of the refugee crisis, on the basis of their respective discourse networks. Our case study, beyond the relevance of its theoretical findings related to the emergence and shaping of coalitions in the debates, serves a two-fold methodological purpose, as well: i) we test the robustness of the network representation by comparing the full dataset to a random sample of it^14^ and ii) we guide the reader through all the steps of tackling research questions with *DebateNet2.0* and mardyR.

Next, in Sect. 5.2, we summarize research conducted on *DebateNet* since its first release in Lapesa et al. (2020). Taken together, the body of work we summarize represents an evaluation of the resource from a two-fold perspective: the usability/reliability of its annotation guidelines, and its potential for political science research in terms of both the breadth of the questions it allows to address and of its use as a basis for the development of NLP solutions to support the complex workflow involved in interdisciplinary projects like the one in which this resource originated.

### Case study: Mediterranean vs. Balkan Route

The combination of claims of different political actors yields a network structure whose analysis comes with its own challenges and chances. On the one hand, the analysis becomes more demanding due to the increased complexity of the task, imposed by inherently existing inter-dependencies (actors may influence each other in their decision to oppose or support claims). On the other hand, this complexity enables a new relational perspective on the discussion. For instance, it permits the researcher to trace how coalitions and alliances are formed or which actors reach out and bridge between hardened fronts.

We exemplify the use case of discourse network analysis by contrasting two distinct time periods of the 2015 debate in this section: the “Mediterranean” versus the later stages of the “Balkan route” (Fig. 5, “limiting immigration”). Firstly, this allows us not only to assess who the driving actors and claims of the discussion at certain time-points are, but, more importantly, also how they relate to each other and form (dynamic) coalitions. Secondly, we compare the networks obtained by using a) the entire dataset of 2015 (*DebateNet2.0*) and b) a previously released subset of the data (*DebateNet1.0*), in order to evaluate how important the size of the dataset is to understand the migration debate at hand.

**Table 7:** DebateNet2.0 over time

Month	# Claims	# Unique categories	# Unique actors	Average degree
January	403	61	160	2.78
February	163	46	79	2.14
March	160	52	75	2.13
April	177	47	76	2.37
May	179	43	65	2.37
June	115	44	59	1.92
July	223	54	95	2.34
August	449	64	175	2.90
September	910	90	247	3.71
October	743	78	239	3.53
November	565	80	164	3.23
December	330	69	109	2.76

#### Network statistics

A first step in the analysis is to describe the network properties on a basic level. Table 7 depicts the number of claim frequencies (second column), the number of unique claim categories (third column), the number of unique actors (fourth column), and the average degree on a monthly basis (last column).

Unsurprisingly, the fluctuating number of claims matches our previous observations (Figs. 2 and 5 ). While September is the month with the most observations, actors and claims, as well as the highest average degree, June operates on a much lower level. Therefore, the intensity of the debate plummets in June, only to increase drastically from August to September. Afterwards it plateaus on a high level until the end of November.

In the following, we once more focus on the time period discussing sea rescue missions in the Mediterranean from 13th April to 31st of May and contrast it with the debate about limiting migration from 28th September to 15th November in conjunction with the Balkan route which ushers in more restrictionist migration policies compared to the preceding “Wir schaffen das” (we can do it) period (see Fig. 5). Additionally, we compare the resulting networks to their counterparts built from the dataset of the previous release (*DebateNet1.0*) with fewer observations but drawn from the same population.

**Fig. 10:**
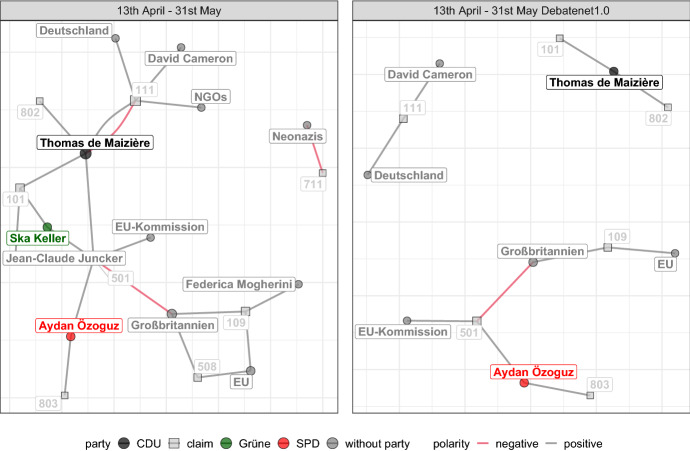
2-slice networks of DebateNet2.0 (left) and DebateNet1.0 (right) from mid April to May

*In the spring,* the core network represents a discussion largely at the EU-level (Fig. 10): The demand to introduce an EU wide solution (501), possibly a quota for the distribution of refugees, is not only at a very central position in terms of degree but also exhibits the highest betweenness-centrality [a measure of how many shortest paths are passing through this node, Freeman (1978)]. Here, it has a brokerage position, bridging two otherwise disconnected components of the network. Similarly prominent is Thomas de Maizière, the minister of the interior, who has a conflicted position with respect to the claim of sea rescue missions (as indicated by the gray and red arc) but supports a EU-wide solution. So do Jean-Claude Juncker and Ska Keller, MP for the Green party. Another relevant claim is that of fighting against people smugglers (109) that finds support from Great Britain (Großbritannien), the EU, and Federica Mogherini (High Representative of the Union for Foreign Affairs and Security Policy of the EU). Within the discussion about fighting against smugglers, it was also suggested to launch a military operation to destroy their boats on the coast of Libya, hence the closed four-cycle with category 508 (military intervention). The national level plays only a small part in the periphery of the network: neo-fascists (Neonazis) opposing Islam (711) which ties in with the islamophobic Pegida movement in Eastern Germany (Patriotic Europeans Against the Islamization of the Occident). Overall, these observations concur nicely with our expectations from the keywords-analysis and proposed time-period: Both topic and actors appear to be largely congruent across methods.

If compared to the network built from *DebateNet1.0* (right panel Fig. 10), the vulnerability of networks (Kossinets, 2006) becomes visible: The core network observed for the complete dataset (left panel, Debatenet2.0) becomes fragmented into components that are no longer connected to each other. By looking at the right panel, one might assume that the actors appear not to talk about the same policies at all. This is of course a question of aggregation and the time windows used. Yet it highlights the importance of a comprehensive analysis: If the task is to analyze the core network of the debate, then the smaller sample would not suffice in this case.

**Fig. 11:**
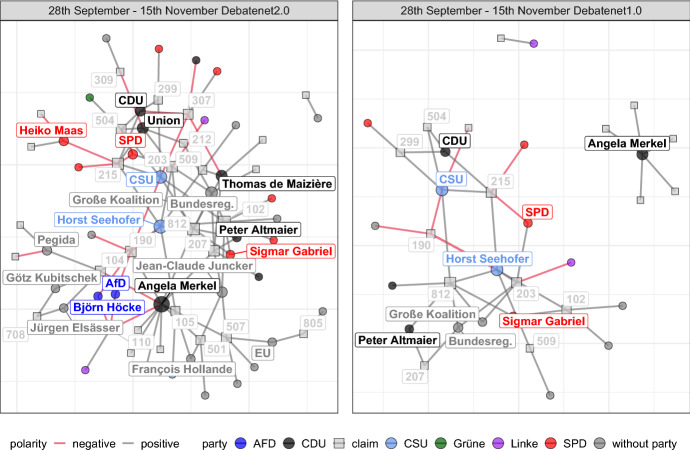
2-slice networks of DebateNet2.0 (left) and DebateNet1.0 (right) from late September to mid November

**Fig. 12:**
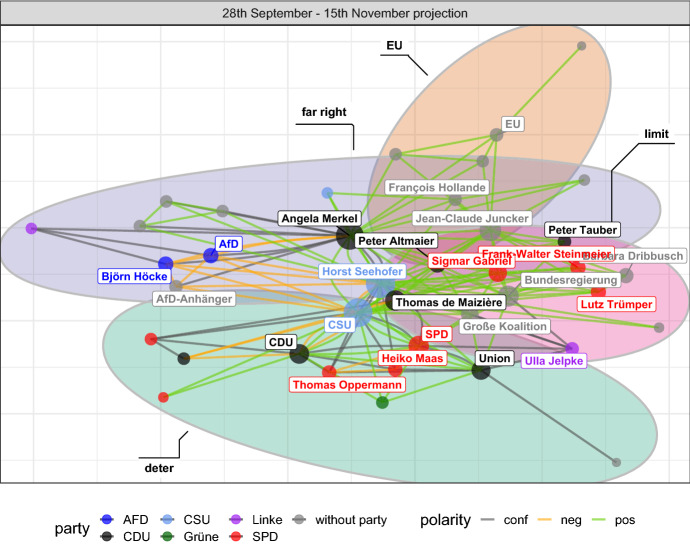
First row: Actor projection of 2-slice network of fall 2015 (Fig. 11)

*In the fall,* this fact becomes even more apparent (Fig. 11). In the left panel, three overlapping clusters within the debate can be observed. The first and largest cluster dedicates itself to questions of residency and integration and involves mostly governmental actors and parties. Arguably, this cluster contains two separate discussions as indicated by the projection onto actor coalitions (Fig. 12): One discussion focuses on deportations (207) and an upper limit (102), while another one discusses so-called transit zones (215) and safe countries of origin (504). While deportations and limits regulate the number of refugees allowed into Germany, transit zones aim to deter applicants by restricting their movement. The second cluster contains the far right movement (e.g. the Alternative for Germany (AfD) and the Pegida movement) and their nationalist claims regarding isolation (104), their expression against the current migration policies (190), and xenophobic and Islamophobic slogans. The third cluster combines actors from the EU and the national level with claims regarding an EU-wide solution (501) via quotas or the readmission-pact with Turkey (507) as well as border controls (105). As foreshadowed by Fig. 8, Merkel has an important bridging function between the different clusters by being connected to many different claims from different clusters in this time period.

However, once we only consider data from the DebateNet1.0, Merkel gets separated from the largest component and the second and third clusters disappear. This indicates that sampling too few articles is unsuitable for the, admittedly highly specific, task of extracting a core network of the migration debate. Nevertheless, the sample is by no means unusable. Instead, it highlights the importance of task-dependency. For example, if the goal is to identify a list of relevant claim categories during the time period from 28th September to 15th November, then even this sample can be considered sufficient: Almost 80 percent of distinct claim categories existent in *DebateNet2.0* are already present in *DebateNet1.0* (74 out of 95). This number increases to 95 percent if the entire year is considered.

*One-mode Projections* of the actors are another way to look at the fall-network that capture co-occurrences within the data (Leifeld, 2016). Recall that actors are linked to each other if they share the same claims and hence form coalitions based on the topical similarity of their political interests (1st row of Fig. 12). Depending on their polarity with regard to the claims in question, the relation may either reflect mutual support, shared disagreement, or conflicting stances. Conceptually, this represents yet another level of abstraction from two-mode representation and its underlying textual information which should make it easier to identify clusters within the network. We do so with a basic community detection algorithm based on (greedy) modularity optimization (Newman, 2004; Clauset et al., 2004) which belongs to the family of (agglomerative) hierarchical clustering procedures and identifies groups of nodes with higher density between them than between nodes of different groups.

The algorithm suggests four co-existing clusters (communities) within the actor-network, shown as colored ellipses in the first panel. The clusters are mostly consistent with the observations made above on the two-mode level: Governmental actors (red, black, and light-blue nodes) are mostly distributed into two clusters (limit and deter, pink and green). European actors are centered around yet another cluster (EU, brown). The last cluster is defined by the debate about fundamental questions of migration by far right actors (far right, violet). The projection approach highlights how dynamic issue communities present themselves as the discourse progresses. For instance, Thomas de Maizière, minister of the interior, now focuses more on domestic policies than he did during the sea rescue discussion earlier in the year (as part of the EU cluster). Moreover, he is densely embedded into the governmental coalition, representing the building of consensus and thus facilitating more decisive actions within the executive. Similarly, Angela Merkel has to defend her earlier “Wir schaffen das” against propositions by the far right AfD, which ultimately would become the biggest opposition party in the 2017 election in Germany.

The benefit of the network-based approach becomes particularly evident by looking at the different kinds of relations expressed in Fig. 12. Without the network perspective, e.g. by looking only at keywords, it would be difficult to infer how different actors relate to each other. This is not to say that keywords do not provide valuable insights on their own. On the contrary, as shown in Table 6 (p 17), relevant actors and claims correspond highly to prominent nodes found in the network (e.g. Thomas de Maizière). In practice, one can initially deploy keyword analysis in order to get a general understanding of the most prominent actors and pressing issues of the debate, followed up by a more detailed DNA approach.

### Further work on DebateNet

We now adopt a broader perspective on the evaluation of *DebateNet2.0* and discuss studies which were based on it or on its earlier version. These provide evidence for the usability and reliability of the annotation guidelines, when applied to subsamples of the data or to different domains/textual sources, as well as the usefulness of the dataset to address political science research questions.

Haunss et al. (2020) contribute to the assessment of *DebateNet1.0* from both the annotation reliability and the workflow support perspectives. They annotate previously unseen articles from the taz corpus using the same guidelines with success. In this experiment, annotators achieve a relatively high reliability when compared to the gold standard, indicating a consistent understanding of the guidelines. On the workflow support side, they target the evaluation of the discourse network extracted with a NLP claim identifier, in a direct comparison to the network extracted with the expert annotation. They find that, due to the natural redundancy present in newspaper articles, the 2-slice network derived from automatically identified claims (with a manual filter on false positives) is essentially equivalent to the one developed from fully manual annotation. In addition, they also show that integrating automatic claim identification in the annotation environment leads to more reliable annotation.

Blokker et al. (2020) test the robustness of the guidelines developed for newspaper articles and successfully expand the domain of its application to party manifestos, which contain an official representation of the position of a party with respect to specific policy issues (Budge et al., 2001), which lends itself well to a comparison to the individual positions of the political actors as reported in newspapers. Conversely, this permits a comparison of the claims made by each actor to the official party line, with possible inferences about the actor’s ideological standpoint as well as about the influence of newspaper report on subsequent party programs (Blokker, 2022).

Furthermore, Yu and Fliethmann (2022) exploit the claim annotation in *DebateNet1.0* to gather an domain-specific vocabulary that they employ for the analysis of the framing of the discourse of the refugee crisis in German newspapers.

## Conclusion

In this paper, we have introduced *Debatenet2.0*, a dataset containing expert annotation on the German political debate on the so-called “refugee crisis” in 2015. The core units of our annotations are political claims (e.g., “quotas for refugees should be established”) and the actors who made those claims (e.g., “Angela Merkel”, “the government”, “the protesters”). While *Debatenet2.0* naturally lends itself to various quantitative investigation approaches (e.g., keyword analysis), its primary purpose is to support a Discourse Network Analysis (DNA) of the debate.

Our contributions comprise multiple levels. At the level of resources, we release *Debatenet2.0* and its companion R package mardyR, which permits the user to create independent queries and visualizations. At the methodological level, we provide a step-by-step explanation of the task of deriving discourse networks from textual data, helping the reader to bridge the conceptual gap between the two fields involved in this interdisciplinary research (political science and computational linguistics). A further contribution is the case study, in which we address the research questions of a) how specific, concrete events in the crisis affected the discourse network representation of the policy debate and b) how data-dependent the discourse network representation is (we compare networks built from the full dataset to those of a subsample). Broadly, we demonstrate how the debate gained momentum by comparing the spring network with the fall network and we highlight the formation of issue communities. Moreover, the vulnerability of the core-network in the face of incomplete (sampled) data is addressed. Beyond answering the topical research questions, our case study serves also as an illustration of the application of DNA, and can serve as a guideline for the formulation of research questions on *Debatenet2.0* (and comparable datasets) and for the interpretation of the results.

Beyond its use to address political science research questions (cf. discussion in Sect. 5.2), *Debatenet2.0* also has a clear potential for method development in NLP, which we have only hinted at so far. DebateNet served as textual basis for the development of models for claim detection and classification which initially used a relatively vanilla transformer-based classification architecture (Padó et al., 2019). However, analysis of these models soon revealed the need for more in-depth research.

First, it was found that these classifiers learn to exploit the fact that some actors very frequently co-occur with claims, and consequently perform poorly on claims by infrequent actors. This is both an issue of fairness (as obviously minorities tend to occur with lower frequency than mainstream actors) and robustness, because unseen data are very likely to have a different actor distribution—in particular when they come from a time period $t+1$ different from the training time *t*. Consequently, Dayanik and Padó (2020) show that masking actor mentions in the training data (or alternatively, adversarial training) leads to better performance on claims of low frequency actors and also improves performance in a cross-domain setting.

Second, it was found that the vanilla classifiers did not perform well on the over 100 fine-grained claim categories since most of these are rare. At the same time, reliable recognition of these categories is crucial to the construction of the discourse network. As before, this problem is both one of fairness and of robustness over time: as shown in our experiments, specific historical events trigger and shape the debate, making a claim infrequent at time *t* (the training data) extremely relevant at time $t+1$ (the data to be analyzed). To address this problem (Dayanik et al., 2022) review different approaches that inject knowledge about the hierarchical structure of the codebook into the classifier and find that a simple structured regularization approach is able to substantially improve the predict for infrequent categories.

More generally speaking, from a NLP perspective, the notion of claim is at the core of the argument mining research community (Cabrio & Villata, 2018; Lawrence & Reed, 2019). Indeed, the domain-specific definition of claim put forward in political claim analysis and implemented in our annotations contributes to the characterization of the very notion of claim which is at the heart of recent work in argument mining (Daxenberger et al., 2017; Schaefer et al., 2022). Beyond claims, at the level of annotation layers, we have meanwhile introduced an additional node type, the so-called *frame* node, which encodes the reason provided by the actor to support a claim (e.g., a border fence should not be built because this would violate human rights). Frame nodes in DNA directly map into the argument mining definition of premise/justification which, at together with claims, represent the core of arguments. Indeed, in Blokker et al. (2022), we show that the use of frames does help in getting a better picture of party positioning: for example, both the Green and a right party may support the introduction of wind turbines, but the former would do it out of environmental concerns, and the latter out of economic reasons.

Political claim and frame analysis tap into the argumentative nature of political discourse, which bring together the two “souls” of argumentation, namely persuasion and reason giving (Lawrence & Reed, 2019). An example of its application is, for example, the mining of debate speeches accompanying political campaigns (Visser et al., 2020; Haddadan et al., 2019), also in a multimodal perspective (Mancini et al., 2022).

Current work on *Debatenet2.0* targets the extension of the time frame for the annotation, to include articles from 2005 and 2010, the annotation of new topics (pensions and COVID-19), and the shift towards a multilingual approach (for the COVID-19 debate). Multilinguality is a known challenge for NLP in general, and argument mining in particular (Eger et al., 2018; Toledo-Ronen et al., 2020). Note that multilinguality is not just a technical NLP challenge, but also a hard conceptual challenge for the political science experts whose aim is to compare the debate in different countries and thus have to devise annotation schemas that are expressive enough to paint a fine-grained picture of the national debate, but also general enough to allow for comparisons across countries. A further challenge we are currently tackling is the use of NLP to support the codebook development process in a semi-automatic: so far, the process has been inductive, as we discuss in the paper, starting from a core of theoretically-motivated categories, and integrated as long as annotation proceeds an relevant new categories are encountered. One way in which NLP can be integrated in the process is through methods that allow the mapping of existing codebooks for the same domain but on different textual sources, e.g., manifestos, as in Blokker et al. (2020), or voting applications as in Blokker et al. (2022). Furthermore, NLP methods can be applied on existing annotation to optimize the codebook—e.g., merging or splitting categories based on the similarity of the items they contain.

## Appendix A: Annotation guidelines: example

See Table 8.

**Table 8:** Fine-grained categories: codebook examples and annotation guidelines

102: Limit immigration/upper limit	
– *Explanation*: This claim refers both to limiting the number of refugees by means of a fixed maximum number and to unspecified demands for a reduction in the number of refugees	
– *Example*: “There will be no way around a limit and thus an upper limit for immigration”	
111: Sea rescue	
– *Explanation*: This claim refers to demands for the rescue of refugees in distress at sea	
– *Example*: “The human rights organization is calling on the EU to set up a joint sea rescue operation on the Mediterranean see off the Libyan coast and to create significantly more admission places for refugees”	
504: Safe legal status for country of origin	
– *Explanation*: This claim refers to demands for the extension of the legal status of one or more countries of origin of refugees as “safe”	
– *Example*: “In addition, the legislator must declare more Balkan countries safe third countries to which we can then deport more quickly”	

## Appendix B: Timeline

- Pegida (01/01/2015–15/02/2015): This time period is characterized by claims made around the Islamophobic Pegida (Patriotic Europeans Against the Islamization of the Occident) movement that was founded in October of 2014. Prominent claims and points of discussion of this period are Xenophobia, Islam, and Public Debate (should politicians engage with the movement or categorically refuse communication?).
- Tröglitz (16/02/2015–12/04/2015): This period revolves around the town of Tröglitz in Germany and the proposal of its mayor to accommodate refugees, inciting (violent) protests and threats by far-right sympathizers and subsequently compelling the mayor to resign. Prominent claims and points of discussion of this period are refugee accommodation and violence against migrants.
- Mediterranean (13/04/2015–31/05/2015): During this period, the media increasingly reported on refugees trying to cross the Mediterranean by boat under high casualties. The EU holds an emergency summit and subsequently proposes a quota to distribute the incoming refugees. Prominent claims and points of discussion of this period are EU-solution, emergency sea rescue, and fight against people smugglers.
- Refugee trail (01/06/2015–01/08/2015): While refugees are still trying to reach Europe from the sea, another route increasingly gains the attention of the media: the Balkan route. Hungary starts to build a border fence to fend off asylum seekers. Still, refugees are reaching Germany invalidating the Dublin-III regulation. Prominent claims and points of discussion of this period are (medical and financial) care for refugees, safe countries of origin, and an immigration law.
- Heidenau (02/08/2015–30/08/2015): As a reaction to a planned refugee shelter, the extreme right and its sympathizers riot and refuse access to the shelter. Emerging claims and points of discussion of this period are right-wing extremism, (accelerated) deportations, and Dublin-regulation.
- “We can do it” (31/08/2015–27/09/2015): Angela Merkel declares on 31th August “Wir schaffen das”, Germany and Austria open their borders (4th September) only to temporary enforce border controls again later that month. Prominent claims and points of discussion of this period are EU-solution, refugees welcome, and border controls.
- Limit immigration (28/09/2015–15/11/2015): The “refugees welcome” enthusiasm is followed by a tightening of asylum restrictions and an increasingly tense climate. Prominent claims and points of discussion of this period are upper-limit, walls-up policy, borders controls, and the cooperation with transit countries.
- International solutions (16/11/2015–31/12/2015): This period largely continues the policy of the previous phase with an slight increase in claims regarding foreign policies (the G20-summit took place in Antalya on 15/16. November). Prominent claims are EU-Solution, cooperation with transit countries, and combat causes of flight.

For a more in-depth analysis of the events and the accompanying media-echo refer to Haller (2017).

## Footnotes

1 An earlier version of the dataset was released in Lapesa et al. (2020) as *DEbateNet-mig15* to which we will refer as *DebateNet1.0*.

2 *DebateNet2.0* is available for download as a CLARIN resource at http://hdl.handle.net/11022/1007-0000-0007-DB07-B.

3 The name is an acronym for *Modeling Argumentation Dynamics of Political Discourse* (in R), which is also the name of the research project within which this research was carried out (cf. acknowledgments).

4 Note that DNA is not the only way of querying the rich annotation in the dataset: Sect. 3.5 provides an overview of the core quantitative properties of the datasets, including a keyword analysis.

5 We are currently extending the annotation to the more right-leaning Frankfurter Allgemeine Zeitung (FAZ) in order to compare the discourses represented in both newspapers. However, because of copyright restrictions, we are not able to make the FAZ data publicly available.

6 The complete data set *DebateNet2.0* contains almost twice as many claims and unique actors as the subset contained in *DebateNet1.0*.

7 Party affiliations are either directly annotated, if provided within the newspaper article, or attributed ex post (either manually or by querying Wikidata).

8 In the interest of better readability, we refrain from reiterating the formalization behind discourse networks and their projections, which are described in detail in Leifeld (2016).

9 Haller identifies 10 pertinent events and investigates how they were conveyed in leading German newspapers. Here, we adopted some parts and labels of the detailed classification of this study and transferred them to a much coarser categorization. For a brief summary of each time period, refer to “Appendix B”.

10 Labeled dates symbolizing micro-events are constructed following https://en.wikipedia.org/wiki/Timeline_of_the_European_migrant_crisis#2015 and https://www.sueddeutsche.de/politik/migration-etappen-der-fluechtlingskrise-in-deutschland-eine-chronologie-dpa.urn-newsml-dpa-com-20090101-160831-99-279450.

11 https://shiny.rstudio.com/ and https://rstudio.github.io/shinydashboard/.

12 The app can be accessed through the mardyR package; a brief manual can be found here:http://hdl.handle.net/11022/1007-0000-0007-DB07-B.

13 The current version does not support external datasets. However, we plan to update a future version with this functionality. Prerequisite for this is a similar data format of (actor—claim—polarity) triples.

14 The random sample corresponds to an earlier version of the dataset, *DebateNet1.0* by Lapesa et al. (2020). While this paper is an extension of Lapesa et al. (2020), note that the overlap is limited to the related work section.
